# How music training enhances working memory: a cerebrocerebellar blending mechanism that can lead equally to scientific discovery and therapeutic efficacy in neurological disorders

**DOI:** 10.1186/s40673-015-0030-2

**Published:** 2015-09-04

**Authors:** Larry Vandervert

**Affiliations:** American Nonlinear Systems, Spokane, WA 990205 USA

**Keywords:** Albert Einstein, Central executive, Cerebellum, Internal models, Music therapy, Sequence detection hypothesis, Scientific intuition, Music training-induced voluntary control, Thought dysmetria, Working memory

## Abstract

**Background:**

Following in the vein of studies that concluded that music training resulted in plastic changes in Einstein’s cerebral cortex, controlled research has shown that music training (1) enhances central executive attentional processes in working memory, and (2) has also been shown to be of significant therapeutic value in neurological disorders. Within this framework of music training-induced enhancement of central executive attentional processes, the purpose of this article is to argue that: (1) The foundational basis of the central executive begins in infancy as attentional control during the establishment of working memory, (2) In accordance with Akshoomoff, Courchesne and Townsend’s and Leggio and Molinari’s cerebellar sequence detection and prediction models, the rigors of volitional control demands of music training can enhance voluntary manipulation of information in thought and movement, (3) The music training-enhanced *blending* of cerebellar internal models in working memory as can be experienced as intuition in scientific discovery (as Einstein often indicated) or, equally, as moments of therapeutic advancement toward goals in the development of voluntary control in neurological disorders, and (4) The blending of internal models as in (3) thus provides a mechanism by which music training enhances central executive processes in working memory that can lead to scientific discovery and improved therapeutic outcomes in neurological disorders.

**Results:**

Within the framework of Leggio and Molinari’s cerebellar sequence detection model, it is determined that intuitive steps forward that occur in both scientific discovery and during therapy in those with neurological disorders operate according to the same mechanism of adaptive error-driven *blending* of cerebellar internal models.

**Conclusion:**

It is concluded that the entire framework of the central executive structure of working memory is a product of the cerebrocerebellar system which can, through the learning of internal models, incorporate the multi-dimensional rigor and volitional-control demands of music training and, thereby, enhance voluntary control. It is further concluded that this cerebrocerebellar view of the music training-induced enhancement of central executive control in working memory provides a needed mechanism to explain both the highest level of scientific discovery and the efficacy of music training in the remediation of neurological impairments.

## Background

The preserved brain of Albert Einstein has been extensively studied on the premise that unique neuroanatomical features of his cerebral cortex may reveal the neural substrates of his exceptional mental abilities [1–3]. These studies found various neuroanatomical differences in Einstein’s brain as compared to ordinary controls. Notably, Men et al. [3] found greater thickness of the majority of sub-regions of Einstein’s corpus callosum. Falk [1] found somatosensory enlargements in Einstein’s brain and generally concluded that since music training results in neuroanatomical changes, and since Einstein took violin lessons as a child and continued playing in adulthood, these enlargements were the result of his music training and may have been responsible for his extreme preference for spatial thinking. (Einstein took violin lessons in early childhood from age 6 to 14 years [4, 5] and then continued to regularly play the violin into adulthood.)

Falk’s [1] conclusion that musicians’ neuroanatomical changes are due to music training rather than genetic endowment has been overwhelming supported by research aimed at understanding the promise of neural plasticity in children with neurodevelopmental disorders. For example, Hyde, Lerch, Norton, Norton, Forgeard, Winner, Evans and Schlaug [6], Schlaug, [7] and Schlaug, Forgeard, Norton, Norton and Winner [8] concluded that music training does indeed produce significant, lifelong changes in the neuroanatomy of the cerebral cortex, including in the corpus callosum, and that these changes are most pronounced when training begins in early childhood. Schlaug et al. [8] were quite clear on this point, “Our results show that it is intense musical experience-practice, *not preexisting differences* [italics added], that is responsible for the larger anterior CC [corpus callosum] area found in professional adult musicians” (p. 205).

## Music training, working memory, and the cerebellum

Collectively, the above studies show that music training changes the structure of the brain and, if continued, contributes to maintaining brain plasticity throughout life. But, through what demonstrable cognitive mechanism(s) did music-induced anatomical changes in the brain contribute to producing Einstein’s level of scientific thinking and, at the same time, offer therapeutic approaches to, for example, neurodevelopmental disorders where voluntary control of speech or movement is involved?

Chen, Penhune and Zatorre [9] investigated brain activity in musician and nonmusicians as they engaged in varying complexities of musical activity. They found that music training recruits the prefrontal cortex to extract “higher-order features of a rhythm’s temporal structure” (p. 226) which, they concluded, leads to enhanced working memory in musicians in retrieving and monitoring information, including the anticipation of sequences. Chen, Penhune and Zatorre’s conclusion that music training improves working memory has been strongly supported by George and Coch [10], Schulze and Koelsch [11] and Suárez, Elangovan and Au [12] for many aspects of working memory including its executive, spatial, and tonal functions.

Ito [13] proposed an explanation for how these improvements in the central executive can be understood as the result of the learning of error-driven cerebellar internal models of *mental models* originating in the parietolateral association cortex, “the highest level at which [models of] our internal world is formed” (p. 481). Ito convincingly argued that through repetitions of thought (or practice—according to Ito, the adaptive learning of internal models in thought and movement is identical) these error-driven adaptive cerebellar internal models progressively increase the speed, consistency, and appropriateness of the central executive (and other components) of working memory. Ito’s [13] assertion that the cerebellum learns internal models of central executive processes of working memory is supported by Schmahmann [14, 15] and Thurling, Hautzel, Kuper, Stefanescu, Maderwald, Ladd et al. [16]. Following Ito’s above description of the cerebellar improvement of thought processes, in the case of music training, then, thought and movement models associated with learning to play a musical instrument (necessarily involving the central executive and, for example, spatial, and tonal components of working memory [10–12])^1^ would be sent to the cerebellum where internal models would be learned for adaptive error-correction as practice proceeded, perhaps for a lifetime. And, it should be pointed out that in terms Ito’s [13] description of the learning of internal models in the cerebrocerebellar system, the executive control structure of working memory would incorporate the multi-dimensional rigor (effector, sensory, affective, mental, autonomic) and intense volitional-control demands of music training as an auxiliary template for cognitive, emotional, and motor integration (footnote-1), and, thereby, produce an increase in the speed, consistency and appropriateness of voluntary control.

It is proposed that the forgoing account of how music training improves especially the central executive of working memory through cerebellar internal models provides a common mechanism behind Einstein’s high-level scientific thinking and, at the same time behind the therapeutic effectiveness of music training in neurological disorders. It should be noted that Ito [13] provided the essential arguments for such a proposal, although not involving music training, by suggesting that the cerebellar mechanism of error-driven adaptive control could account for both the highest intellectual thinking (cognitive modeling), and at the same time, if this mechanism did not operate properly, would result in a *dysmetria of thought* (Schmahmann [17]). Moreover, Schmahmann’s [15] detailed description of dysmetria of thought, especially in its effect on central executive and affective processes provides insight into specific cerebrocerebellar processes that can be accelerated toward high levels of achievement in people with domain sensitivities like Einstein^2^ or, if dysfunctional, can result in *an overall lowering of intellectual function*. That is, the cerebellar modulation of the motor, sensory, cognitive, affective and autonomic functional systems normally permits the production of harmonious, integrative behavior of those systems. However, Schmahmann [15] proposed, the loss of the cerebellar component of these same neural circuits produces dysmetria of thought that results in the cerebellar cognitive affective syndrome:

It [the cerebellar cognitive affective syndrome] is characterized by (1) disturbances of executive function, which includes deficient planning, set-shifting, abstract reasoning, working memory, and decreased verbal fluency; (2) impaired spatial cognition, including visual-spatial disorganization and impaired visual-spatial memory; (3) personality change characterized by flattening or blunting of affect and disinhibited or inappropriate behavior; and (4) linguistic difficulties, including dysprosodia, agrammatism and mild anomia. *The net effect of these disturbances in cognitive functioning was a general lowering of overall intellectual function* [italics added]. (Schmahmann ([15], p. 371))

It is suggested that the varied aspects of music training not only enhance, especially the central executive processes of working memory [9–12], but also can enhance to varying degrees all of the foregoing interrelated intellectual and affective components of the cerebellar cognitive affective syndrome (see parallel music training components, footnote-1).

## Purpose

The foregoing introduction provides a framework that describes music training-induced neuroplasticity and the resulting cerebrocerebellar improvement of the central executive functions of working memory. The purpose of this article is to further elaborate this initial framework by describing a detailed cerebrocerebellar mechanism that can explain both the highest levels of scientific contribution (as in the case of Einstein), and, at the same time, the therapeutic effectiveness of music therapy in the treatment of neurological disorders. While Vandervert, Schimpf and Liu [18] offered a preliminary cerebrocerebellar explanation of creativity in scientific advancement, and Altenmuller and Schlaug [19] proposed an explanation involving a positive “transfer effect” of music training on emotional and cognitive functions and thus on neurological disorders, neither of these approaches offered a detailed mechanism that could explain specifically how steps forward in either high-level science or improved therapeutic outcomes might occur as the result of voluntarily controlled thought or movement.

To offer just such a detailed mechanism, this article describes (1) How, in accordance with Akshoomoff, Courchesne and Townsend’s [20] and Leggio and Molinari’s [21] cerebellar sequence detection hypotheses, internal models which are learned in the building of initial central executive processes in the infant’s working memory, (2) How, a music training-enhanced central executive may lead to more intense attentional manipulation in working memory and thus more broadly adaptive cerebrocerebellar blending of internal models (Imamizu & Kawato [22, 23], Imamizu, Higuchi, Toda & Kawato [24] in working memory, and (3) How cerebellar internal models learned in accordance with the cerebrocerebellar blending process described in (2) above can give rise to unconscious intuition (solutions) in both scientific discovery and in the therapeutic efficacy of music training.

## The cerebrocerebellar building of central executive processes in the working memory of the infant

According to Baddeley’s [27] model of working memory the central executive is an attentional control system which shifts, divides, and focuses attention among visual-spatial, phonological and long-term memory information. To adequately understand the cerebrocerebellar foundations of the central executive processes of working memory it will be necessary to examine the earliest beginnings of the development of the central executive in the infant. This will allow us to connect attentional control in the central executive with a cerebrocerebellar explanation.

The most detailed behavioral research in which the infant’s initial foundations of the attentional processes of working memory are clearly revealed is that of Mandler [28–31]. Mandler proposed that the infant’s attention-driven *repetitive* perceptual-motor tracking of its own bodily movement and objects moving in the environment (the relationships among objects, space, and time) is *“distilled”* or *“condensed”* [29] into *conceptual primitives*. She referred to this process as *perceptual meaning analysis* [30]. Mandler [29] further proposed that these conceptual primitives provide the bases for both simple inferential and analogical thought and a basis for the acquisition of the relational aspects of language in the infant. Since Mandler proposed that perceptual meaning analysis in the infant is the result of, “an *attentional mechanism* [italics added] dedicated to simplifying spatiotemporal information” (([31], p. 426)), and that it thereby provides the bases for both spatiotemporal and language manipulation, Vandervert [32–34] suggested that what she refers to as perceptual meaning analysis actually describes, in Baddeley’s [27] model, the birth of the central executive of working memory and its initial establishment of the visual-spatial sketchpad and the mental framework for the phonological loop. Vandervert proposed that since the foregoing distillation of the infant’s movements and perceptual-motor tracking are attention-driven and highly repetitive, working memory is brought into functional use and constantly improved by adaptive error-driven cerebellar internal models as originally described by Ito [13] and as more recently described by Akshoomoff, Courchesne and Townsend [20] and Leggio and Molinari [21].

**Fig. 1:**
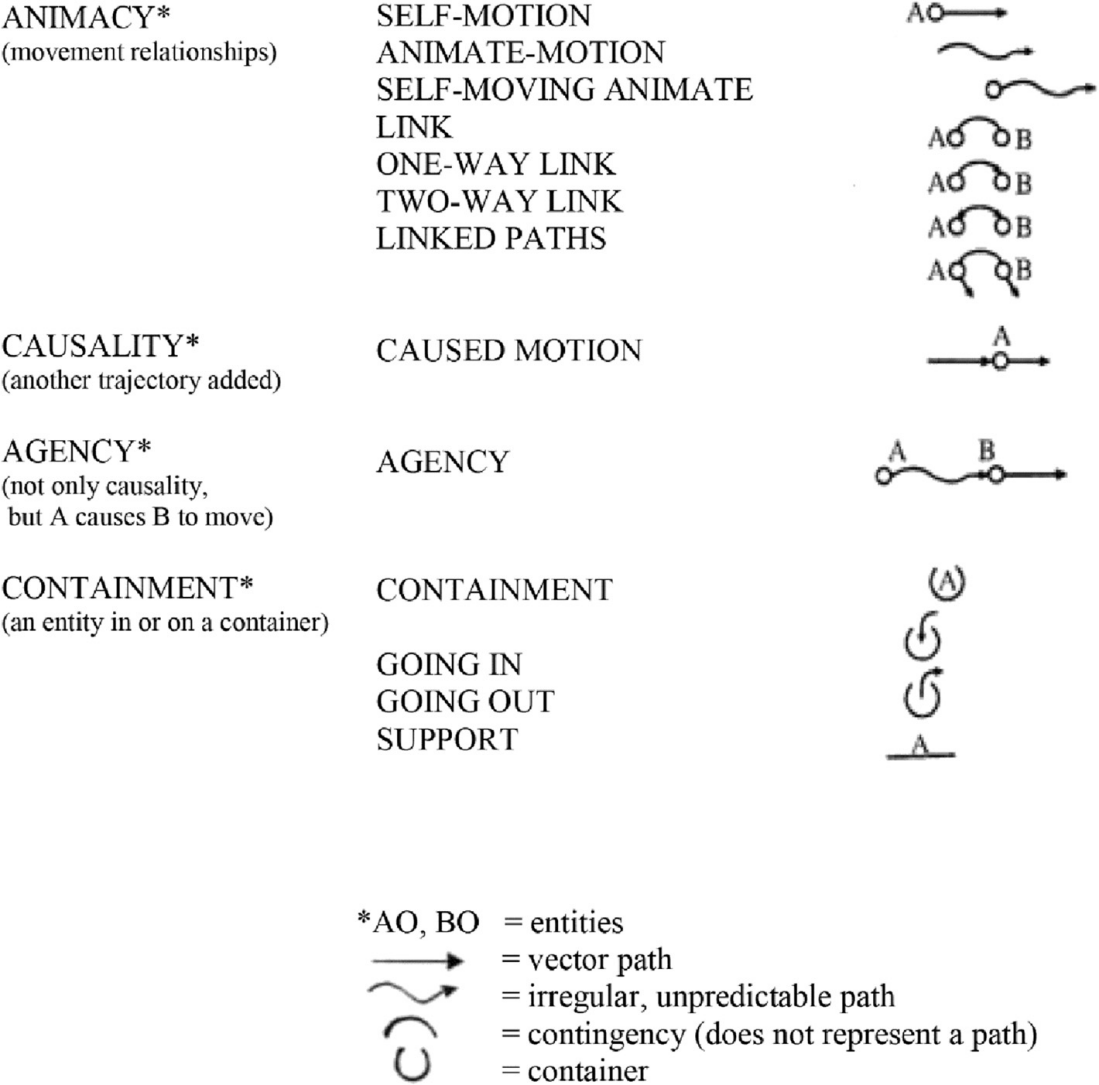
Mandler’s [7, 8] conceptual primitives—collectively, the infant’s unconscious “primitive physics”

Figure 1 illustrates Mandler’s characterizations of the “spatiotemporal” conceptual primitives she derived from her extensive experiments with infants^3^.

According to Mandler [31], the conceptual primitives shown in Fig. 1 result from the infant’s perceptual meaning analysis and thereby form the foundational basis of *consciously accessible visuospatial meanings* to which later symbols used in relational thought and language refer. As proposed in this article, these conceptual primitives were derived from the earliest moments of the operation of the central executive attentional patterning as it established adaptive (and error-driven) working memory including accessible “slave” components (a la Baddeley [27]) in Fig. 1, which, altogether, permitted simple inferential and analogical thought—see Mandler [29] above.

## The constant improvement of central executive control through cerebellar internal models: more details

Vandervert [34, 35] argued that Mandler’s conceptual primitives are encoded as cerebellar internal models in the infant’s working memory in accordance with Akshoomoff, Courchesne and Townsend’s [20] and Leggio and Molinari’s [21] cerebellar sequence detection and prediction models. Within this framework of the infant’s developing working memory, it is proposed that the cerebellar sequence detection hypothesis proposed by Leggio and Molinari [21] describes the details of the learning of the internal models by which the central executive builds the initial visual-spatial working memory:

According to this hypothesis, the cerebellum detects and simulates *repetitive patterns of temporally or spatially structured events* [italics added], regardless of whether they constitute sensory consequences of one’s actions in motor planning, expected sensory stimuli in perceptual prediction, or inferences of higher-order processes (e.g., *cognitive elaboration* [italics added] or social cognition). The simulation allows internal models to be created that can be used to make predictions about future events that involve any component, such as the *body, other persons, and the environment* [italics added]. (p. 36)

Leggio and Molinari’s above description comports strikingly well with both Mandler’s [29–31] description of the formation of the conceptual primitives in Fig. 1 and with Vandervert’s [34, 35] proposal that these conceptual primitives are formed through the learning of cerebellar internal models which allow the prediction of future patterns of sequences, for example in typing or in learning to play a musical instrument.

That the infant cerebellum encodes precisely such body/environment predictive internal models as originally proposed by Vandervert [32, 33, 36] is supported by the following recent, converging research. First, visual-spatial working memory begins to be established in the infant by six months of age [37]. Second, Short, Elison, Goldman, Styner, Gu, Connelly et al. [38] found that the growth of neural networks for working memory in the infant are the same as those in older children and adults in connecting frontal, parietal and temporal regions of the brain. Moreover, strongly supporting Short, Elison, Goldman et al. [38], Knickmeyer, Gouttard, Kang, Evans, Wilber, Smith et al. [39] argued that the 240 % increase in the size of the cerebellum in the first year suggested the early involvement of the cerebellum in the formation of the infant’s central executive and visual-spatial working memory:

Because the cerebellum is critically involved in motor coordination and balance [40] the striking cerebellar growth may underpin the rapid motor developments of infancy. The cerebellum has also been implicated in a plethora of other cognitive abilities including planning, set-shifting, language abilities, abstract reasoning, *working memory* [italics added], and *visual-spatial organization* [italics added] [41]. Given that “cognitive” regions of the cerebellum have reciprocal projections with nonprimary frontal, parietal, and occipital association cortex [42], the extremely rapid growth of the cerebellum in the first year may be a prerequisite for specific aspects of later cortical development. (([39], p. 12180))

To further support this argument it is suggested that the *transition* from visual-spatial working memory toward unconscious inner speech in the infant’s early-developing verbal working memory draws upon the premotor cortex, pre-SMA and superior cerebellum (Lobule VI and Crus I) (Marvel & Desmond, [43, 44]). This contention is strongly supported by Liao, Kronemer, Yau, Desmond and Marvel [45] who subsequently found that, indeed, nonverbal (pictorial) information draws upon these same motor regions. Thus, again, Mandler’s proposal that later, consciously accessible language concepts are built from the infant’s visual-spatial conceptual primitives (Fig. 1) and Vandervert’s proposal that these conceptual primitives are formed through the learning of cerebellar internal models in what is becoming the infant’s working memory therefore squares well with Knickmeyer, Gouttard, Kang, Evans, Wilber, Smith et al.’s [39] suggestion that the unparalleled 240 % increase in the size of the cerebellum in infancy is a prerequisite for the later cognitive development of specific regions of the cerebral cortex

## Einstein’s strong view on unconscious intuition

Now, we can connect the above-described cerebrocerebellar foundations of the central executive of working memory with how cerebellar internal models produce unconscious steps forward ("intuition") in the highest levels of scientific discovery. Einstein was one of the clearest thinkers of our time, and in addition to deep thought about theoretical physics, he often probed the psychology of his own imagination. Among his conclusions from these deep personal reflections was that new principles in science could only come about through unconscious intuition. It should be recognized that the acceptance of a vital role of intuition in science is quite common among famous scientists, including Poincare and many Nobel laureates (Shavinina, ([46], esp. pp. 661–663)).

In his classic 1936 article, *Physics and Reality*, where he talked about a variety of psychological and epistemological issues, Einstein was adamant that, “The connection of the elementary concepts of everyday thinking with complexes of sense experiences can only be comprehended intuitively and it is unadaptable to scientifically logical fixation,” (([25], p. 351)), and over a decade later in 1949, he emphasized this same strong view in a personal letter to fellow physicist H.L. Gordon:

A new idea [a new concept] comes suddenly and in a rather intuitive way. *That means it is not reached by conscious logical conclusions* [italics added]. But thinking it through afterwards you can always discover the reasons which have led you *unconsciously* [italics added] to your guess and you will find a logical way to justify it. Intuition is nothing but the outcome of *accumulated earlier intellectual experience* [italics added]^4^.

In his Autobiographical Notes, Einstein [26] recounted perhaps the seminal point of formulation of his special relativity theory as he pictured it in the form of a paradox within his working memory:

After ten years of reflection such a principle [one that would correct errors in existing physical theory] resulted from a paradox upon which I had already hit at the age of sixteen: If I pursue a beam of light with the velocity *c* (velocity of light in a vacuum), I should observe such a beam of light as a spatially oscillatory electromagnetic field at rest. However, there seems to be no such thing, whether on the basis of experience or according to Maxwell’s equations. From the very beginning it appeared to me intuitively clear that, judged from the standpoint of such an observer, everything would have to happen according to the same laws as for an observer who, relative to the earth, was at rest. For how, otherwise, should the first observer know, i.e., be able to determine, that he is in a state of fast uniform motion?

One sees that in this paradox the germ of the special relativity theory is already contained. (Einstein [26] p. 53)

## Extending the cerebellar sequence-detection and prediction to unconscious intuition: cerebrocerebellar blending

But how, exactly, is Einstein's above speed-of-light paradox to be resolved within a *wholly new* contrivance of space-time in accordance with Leggio and Molinari's [21] cerebellar sequence detection and predictive adaptation and accomplish it on the basis of the spatiotemporal foundations (Fig. 1) of the central executive of working memory? Imamizu, Higuchi, Toda and Kawato [24], Imamizu and Kawato [22] and Imamizu and Kawato [23] have shown that when people are confronted with new situations the learning of cerebellar internal models is modified in ways, which although attention-driven, produce solution outcomes unfamiliar to the learner. Specifically, these researchers concluded that when confronting *new* situations, subjects used *blends* of internal models already learned when a new problem was presented to them. It is proposed that these solution outcomes that are unfamiliar to the learner are what is traditionally called “intuition.” Imamizu et al.’s [24] research thus provides valuable insight into how, through cerebellar blending, central executive control in working memory might “elaborate” toward intuition in extended cases where the scientist (or anyone) is struggling with the development of new ideas or new technology. It is further proposed that this same internal-model blending mechanism resulting in intuition would of course apply to the child or adult who, through central executive control, is struggling to forge new movement or thought capabilities in the face of neurological impairment. This notion of the elaboration of central executive processes is in agreement with Leggio and Molinari’s [21] cerebellar sequence detection hypothesis involving “inferences of higher-order processes (cognitive elaboration)” (p. 36). (See above description of “sequence detection hypothesis.”) Additionally, as to elaboration of central executive processes in those with neurological disorders, this contention is strongly supported by “forward model mapping” found in cerebellar-premotor circuitry of practiced pianists which integrates (blends or binds) visual, auditory, and tactile information [47].

Moreover, and particularly salient to Einstein’s above 186,000-miles-per-second, paradoxical imagery in working memory, Yomogida, Sugiura, Watanabe, Akitsuki, Sassa, Sato, et al. [48] found critical involvement of the cerebellum in mental visual synthesis. These researchers defined mental synthesis as follows:

Mental visual synthesis is the act or power of forming a mental image of something not perceived by the senses or present in reality. It consists of taking parts of our various conceptions and combining them to give new forms and images more selective, more striking, more delightful and more terrible, among other things, than those existing in reality. (([48], p. 1376))

Yomogida et al.’s definition of mental visual synthesis includes precisely the type of imaginative visual synthesis involved in, for example, Einstein’s above classic contemplation of the possible consequences of traveling parallel to (“chasing”) a beam of light at 186,000 miles per second. Following Leggio and Molinari’s sequence detection hypothesis [21], it seems clear that such a speed-of-light scenario would seem paradoxical (to Einstein or anyone) from the predictive standpoint of the internal models depicted in Fig. 1. The point here, according to Imamizu, Higuchi, Toda and Kawato [24] and Yomogida et al. [48], is that if one attempts to imagine or manipulate a problem in new ways, new, hybrid (blended) cerebellar internal models develop which will predict newly hybridized visual-spatial configurations, such as, in Einstein’s case, relativity theory. However, this is not to say that such new hybridized scientific hypotheses, will not encounter problems related to validation^5^. Again, as suggested above, this same blending of internal models would apply when one with neurological disorders struggles with new speech or movement capabilities.

In passing, it should be noted that Yomogida Sugiura, Watanabe, Akitsuki, Sassa, Sato, et al. [48] did not mention cerebellar internal models in their study. This omission is largely due to their focus on investigating only the roles of areas of the cerebral cortex in visual synthesis. However, the tabled data and brain images presented in their article importantly implicated the involvement the cerebellum, and therefore should be re-visited with a new focus on the specific roles of cerebellar internal models in regard to Imamizu, Higuchi, Toda and Kawato’s [24] findings.

## The moment of unconscious intuition: the respective roles of cerebellar internal models and working memory (the mechanism)

But how, exactly, are things orchestrated in the cerebellum and cerebral cortex so that intuition arising from the blending of internal models materializes in consciousness to produce new ideas in science? Ito [49] proposed how intuitive advances in both motor and mental control would take place at the unconscious level in the cerebellum and then suddenly enter the consciousness of working memory. Ito used the cerebellum’s forward and controller models to provide the following example of how unconscious intuition would suddenly enter consciousness:

If the forward and inverse [controller] model controls are combined, an interesting possibility emerges [after much practice or contemplation] that the …cerebellum conducts the entire process of thinking … which will not come up to the level of consciousness. This may explain our daily experience that, after repeated trials of learning, a correct answer [or a correct movement] pops out readily without a conscious effort. (([49], p. 102))^6^

It is proposed that within Ito’s foregoing unconscious, “entire process of thinking,” cerebellar *blending* [24, 48] goes on among forward and controller internal models, and thereby produces intuition. The mechanism of the blending of internal models apparently knows no limits to “creative” adaptive error correction. Imamizu, Higuchi, Toda, and Kawato [24] argued that cerebral blending of multiple cerebellar cognitive-manual skill routines and strategies bestowed several tightly interrelated advantages: (1) Interference between different learning epochs is reduced thereby enabling the rapid switching of sequential skilled behaviors, (2) Entirely new skill demands can be coped with by adaptively blending pre-existing motor and cognitive primitives as multiple cognitive-manual skill routines and strategies, (3) Multiple cognitive-manual skill routines and strategies are blended *in proportion* to the requirements of the current new context, and (4) Because blending is proportionate to the specific requirements of changing contexts, an enormous, perhaps limitless, repertoire of behavior can be generated even when the number of cognitive-manual skill routines and strategies might be limited. All four advantages of the adaptive blending of internal models offer insight into how music training-induced enhancement of central executive processes (attentional control) produce steps forward in therapies based on music training.

As Einstein [25, 26] commented (earlier in this article) on his own intuitions, this intuitive “popping out” of a correct (or at least possibly correct) answer or solution “comes suddenly and in a rather intuitive way” (letter to H. L. Gordon, Footnote-4). At the same time, as Ito [49] suggests in the above quote, intuitive insight is a fairly common part of “daily experience”—although these daily intuitions are usually “small” intuitions of little importance. In this regard, it is suggested that everyday intuitions, larger scientific intuitions, and intuitive steps forward that occur during therapy in those with neurological disorders all operate according to this same mechanism of the adaptive, error-driven blending of cerebellar internal models.

## Conclusions and Discussion

In accordance with Akshoomoff, Courchesne and Townsend’s [20] and Leggio and Molinari’s [21] cerebellar sequence detection and prediction models, the cerebellum is constantly learning adaptive internal models related to, among other functions, the prospective goals of working memory [20]. Within these sequence detection models the cerebellum constantly updates adaptive predictions of the consequences of thought and movement. From infancy, the entire framework of the volitional/executive structure of working memory is a product of the cerebrocerebellar system which can incorporate the multi-dimensional rigor (effector, sensory, affective, mental, autonomic) and volitional-control demands of music training as an auxiliary template for cognitive, emotional, and motor integration (footnote-1), and, thereby, for voluntary control. It is concluded that this cerebrocerebellar view of the music training-induced enhancement of central executive control in working memory provides a needed mechanism to explain the efficacy of music training in the remediation of neurological impairments. And, it is suggested that this enhancement of central executive control operates in parallel with Altenmuller and Schlaug’s [19] proposed positive “transfer effect” of music training mentioned in the Purpose section of this article.

In the sequence detection and prediction process the cerebellum encodes temporally ordered sequences of information and as similar sequences later unfold, “they elicit a readout of the full [error-corrected] sequences in advance of the real-time events,” thus, “in contrast to conscious, longer time-scale anticipatory processes mediated by cerebral systems, output of the cerebellum provides moment-to-moment, unconscious, very short time-scale, anticipatory information (Akshoomoff et al. ([20], p. 592)).” This sequence detection process allows central executive processes of working memory to gain a combination of enhanced unconscious (cerebellar) and voluntary predictive control in, for example, playing a musical instrument. In addition, however, within this cerebrocerebellar framework, cerebellar internal models are also adaptively blended within working memory [22–24, 48]. This blending of internal models, it is proposed, provides a second level of adaptive error-correction that is virtually unlimited in its capacity to combine predictive cognitive and motor information in advance and in newly adaptive ways. “Intuition,” as defined in this article, is a sudden updated, predictive solution toward obtaining a prospective goal in working memory that is brought about through the unconscious cerebrocerebellar blending of internal models. (See also, “The Moment of “Intuition’” in Vandervert, Schimpf and Liu ([18], p. 14).) It is further concluded that this error-driven blending of cerebellar internal models is the mechanism behind both the highest levels of scientific discovery and the efficacy of therapeutic approaches to the remediation of neurological impairment.

## Footnotes

1 Music training, by the very nature of its cognitive, affective and manual production requirements, draws heavily on the components and capacities of working memory. Broadly, working memory is the capacity to temporarily maintain and subsequently manipulate information “online” in the pursuit of goal-oriented tasks (see, for example, Baddeley [27]). Thus, within working memory, playing an instrument such as the violin, as did Einstein, requires the engagement of many simultaneous online, goal-oriented mental/production tasks. These working memory tasks include reading the musical notation system and sustaining the highly attentive repetitious practice required to learn the transfer this notation into sequences of bimanual production, the latter of which depends on multisensory feedback. This bimanual production requires high, prolonged attention to developing fine motor skills that is in-sync with metric precision running through the memorization of complex and lengthy musical passages. Added to this is the “on-the-fly” task of emotion-driven improvisation, the limits of which executive and affective processes must closely monitor. (The foregoing description of the components of music training was paraphrased from Wan and Schlaug ([49], pp. 566–567)).

2 Of course not all who undergo music training become an Einstein or a child prodigy. The effects of music training toward such extreme levels of development depends upon the individual’s initial sensitivity (Shavinina [50]; Vandervert [32]) and intensity of early and long-term training, for example, the long-term, dedicated training such as that of deliberate practice as espoused by Ericsson, Roring and Nandagopal [51] and Ericsson [52].

3 It must be noted that the illustrations in Fig. 1 are meant only to convey analogies of the relationships among space, time, and objects and not meant to depict any particular actual experiential components or images in working memory. However, at the same time, since these conceptual primitives are *meanings* [30, 31] from which later concepts may be built or bound (mixed or blended), these conceptual primitives permit/guide the *possibility* of fully conscious manipulation in working memory. See Gilchrist and Cowan [53] for the involvement of underlying *unconscious* processes in working memory, such as the manipulation of conceptual primitives.

4 Albert Einstein letter to Dr. H. L. Gordon, May 3, 1949. This is Item 58 -217 in the Control Index to the Einstein Archive which may be consulted at Mudd Library, Princeton University.

5 Readers may contact the author on epistemological issues related to how, within this model of unconscious intuition, Einstein argued that science maintains connected to empirical validation in the “real world.”

6 This unconscious “thought” process in the cerebellum is a strong conviction of Ito’s. Ito [54] followed up on the 2005 quote by providing a more detailed explanation for how models in the cerebellum might give rise to new ideas that might suddenly “pop-up” in consciousness. In passing, it should be noted that the idea that intuition arises from unconscious models computed in the cerebellum was proposed independently in parallel to Ito by Vandervert [55–58] and Vandervert, Schimpf and Liu [18] in relation to creativity and innovation.
